# Silibinin promotes hepatocyte proliferation through PINK1/Parkin-mediated mitophagy to alleviate acetaminophen-induced liver injury

**DOI:** 10.1007/s10238-026-02218-z

**Published:** 2026-07-03

**Authors:** Fengming Xu, Mohamed Albadry, Olaf Dirsch, Uta Dahmen

**Affiliations:** 1 Department of Infectious Diseases, The First Affiliated Hospital of Zhejiang Chinese Medical University, Hangzhou, 310006 China; 2 Experimental Transplantation Surgery, Department of General, Visceral and Vascular Surgery, Jena University Hospital, 07747 Jena, Germany; 3 Else Kröner Graduate School for Medical Students “JSAM”, Jena University Hospital, 07747 Jena, Germany; 4 Department of Pathology, Faculty of Veterinary Medicine, Menoufia University, 6131567 Shebin El Kom, Egypt; 5 Institute for Pathology, BG Klinikum Berlin, 12683 Berlin, Germany

**Keywords:** Silibinin, Hepatocyte Proliferation, Autophagy, Mitophagy, Acetaminophen-Induced Liver Injury

## Abstract

Acetaminophen (APAP) intoxication is a common cause of liver injury. Silibinin has demonstrated potent hepatoprotective properties. However, its underlying mechanisms in APAP-induced liver injury (AILI) remain unclear. Autophagy is a critical adaptive response in AILI, contributing to the clearance of damaged mitochondria and the attenuation of oxidative stress. Therefore, we focused primarily on investigating the role of autophagy in mediating the hepatoprotective effects of silibinin. The effects of silibinin were evaluated in both AML12 cells and a C57BL/6J mouse model of AILI. Both the in vitro and in vivo experiments comprised four groups: a control group, an AILI model group, a silibinin treatment group, and a silibinin plus autophagy inhibitor group using PINK1-siRNA in cell culture and 3-Methyladenine in the animal experiment. Following induction of the AILI model in mice with APAP at a dose of 300 mg/kg, the animals received the designated interventions for five consecutive days. Histopathological alterations were assessed using hematoxylin–eosin staining. Hepatocyte proliferation and apoptosis were evaluated using the CCK-8 assay and immunohistochemical staining for Ki-67 and cleaved caspase-3, respectively, as well as ELISA for Cyclin D1. Liver function was assessed by serum biochemical analysis of alanine aminotransferase, aspartate aminotransferase, total bilirubin, and albumin. Mitochondrial oxidative stress–related parameters, including superoxide dismutase and malondialdehyde, were measured using colorimetric assays. The expression of autophagy-related genes and proteins (PINK1, Parkin, AMPK, LC3 and p62) was analyzed by quantitative PCR, immunofluorescence, and Western blotting. Transmission electron microscopy was employed to examine mitochondrial ultrastructure and the formation of autolysosomes in mouse liver tissue. In AML12 cells, silibinin mitigated AILI by activating the PINK1/Parkin pathway, thereby promoting mitophagy and enhancing cell proliferation. Co-treatment with autophagy inhibitor PINK1-siRNA attenuated these protective effects of silibinin. In AILI mice, silibinin treatment markedly improved liver function, attenuated inflammatory responses, restored mitochondrial function, and enhanced hepatocyte proliferation. These improvements were associated with increased LC3-II expression and reduced p62 accumulation, indicating enhanced autophagic activity. Notably, the protective benefits of silibinin were significantly attenuated by the autophagy inhibitor 3-Methyladenine. Our findings suggest that silibinin protects against AILI by activating PINK1/Parkin-dependent mitophagy, which mitigates oxidative stress and inflammation while promoting hepatocyte regeneration.

## Introduction

Acetaminophen (APAP) intoxication is one of the leading causes of acute liver injury worldwide [1, 2]. In the United States alone, APAP overdose results in approximately 82,000 emergency department visits annually, making it a primary cause of both acute liver injury and liver failure, accounting for around 50% of all acute liver failure cases [3]. Although N-acetylcysteine (NAC) is commonly used as a treatment for Acetaminophen-induced liver injury (AILI) [4], it is associated with a range of adverse effects, including a narrow therapeutic window, gastrointestinal intolerance, and anaphylactoid reactions [5–7]. More importantly, prolonged treatment with NAC may impair liver regeneration following acetaminophen-induced hepatotoxicity [8]. In the early stages of acetaminophen overdose, treatment with NAC can be considered to counteract hepatotoxicity. However, in the later stages, when extensive hepatocyte death has occurred, effectively promoting hepatocyte proliferation may play a crucial role in improving patient outcomes. Given the limitations of NAC, there is a clinical need for safer, more flexible, and effective therapeutic options. Therefore, exploring alternative compounds with hepatoprotective potential, especially those that can enhance hepatocyte regeneration, is of great importance.

Silibinin, a natural flavonoid extracted from Silybum marianum, has gained attention for its substantial hepatoprotective effects and excellent safety profile [9, 10]. Traditionally used to treat various liver diseases such as chronic and acute hepatitis and cirrhosis [11–14], silibinin has been shown to exhibit strong antioxidant and anti-inflammatory effects [15, 16]. Although these properties have been widely studied, the specific mechanisms by which silibinin confers protection against acute liver injury, particularly AILI, remain largely unclear.

Recent studies have shown that mitochondrial dysfunction plays a critical role in AILI [17]. Mitochondrial dysfunction is characterized by impaired oxidative phosphorylation, loss of mitochondrial membrane potential, excessive reactive oxygen species (ROS) generation, and disrupted energy metabolism [18]. These alterations ultimately lead to the opening of the mitochondrial permeability transition pore, the release of cytochrome c, and the activation of apoptotic signaling cascades [19, 20]. Autophagy is an important cellular mechanism for recycling intracellular components. During autophagy, dysfunctional organelles, misfolded proteins, and invading pathogens are degraded through the lysosomal pathway into basic components such as amino acids, nucleotides, and fatty acids, which are then reused by the cell [21]. Autophagy promotes cell survival by helping cells adapt to environmental stressors such as nutrient deprivation, hypoxia, and toxin exposure. Mitophagy, a selective form of autophagy, plays a pivotal role in clearing damaged mitochondria, promoting mitochondrial regeneration, maintaining energy homeostasis, and supporting cell survival under stress [22]. The division and growth of hepatocytes require a sufficient energy supply, as highlighted in our previous study [21, 23]. Therefore, modulating hepatic autophagy, particularly mitophagy, may provide a novel therapeutic approach for the prevention and treatment of AILI. Previous studies have highlighted the importance of the PINK1/Parkin pathway-mediated mitophagy as a key regulator of mitochondrial quality control [24, 25]. However, whether the protective effect of silibinin is mediated by activating this pathway to alleviate AILI has not yet been explored.

This study aims to investigate whether silibinin activates PINK1/Parkin pathway–mediated mitophagy to improve mitochondrial quality control in hepatocytes. We further examine whether this mechanism reduces oxidative stress and inflammation, promotes cell survival, and promotes hepatocyte proliferation in the context of AILI. By utilizing both in vitro and in vivo models, we aim to gain deeper insights into how silibinin can effectively mitigate AILI and explore the underlying mechanisms. Ultimately, our findings may provide new theoretical insights for the development of more effective therapeutic strategies for treating AILI.

## Materials and methods

### Experimental design

**Fig. 1:**
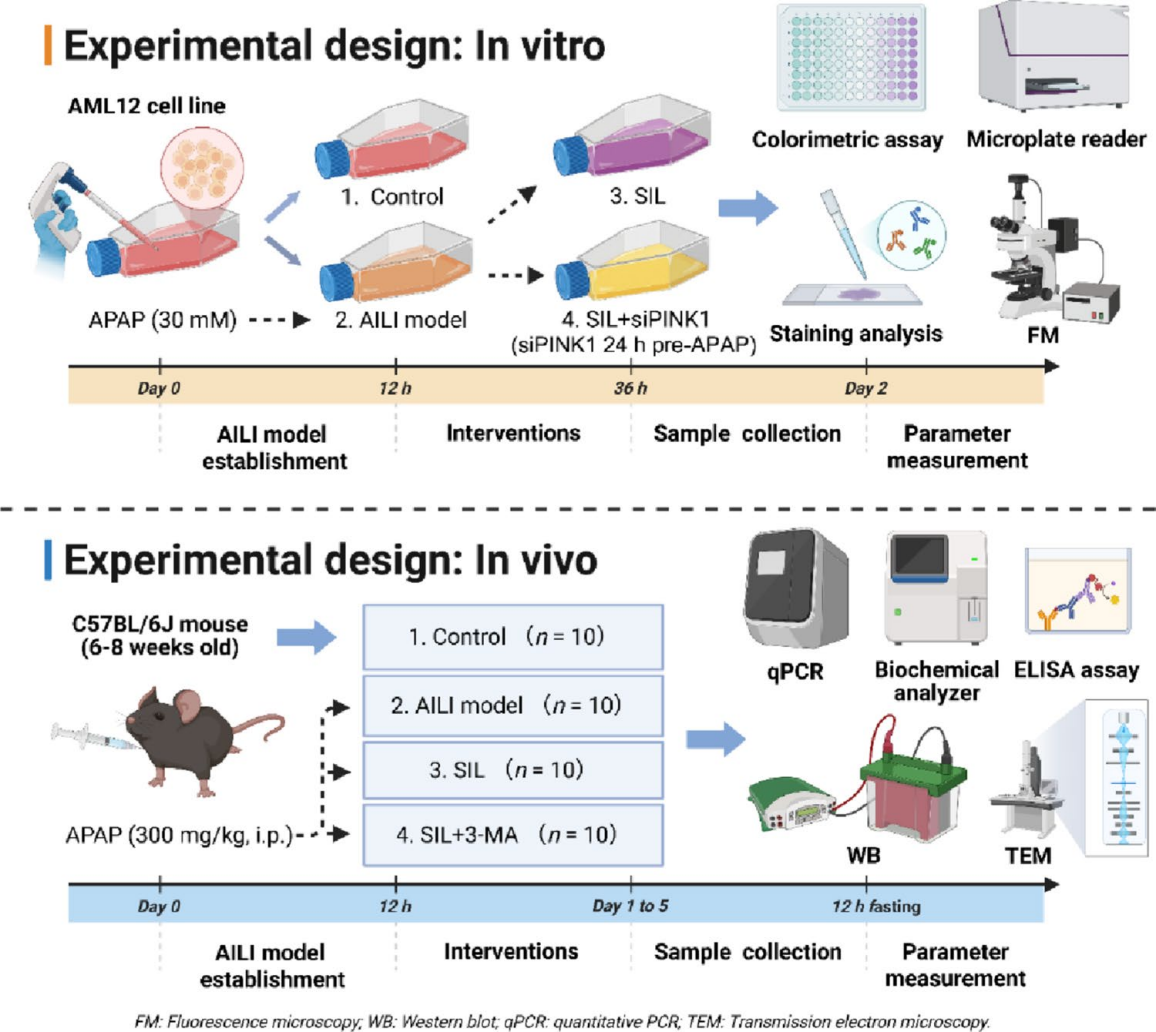
Schematic diagram of the experimental design. In vitro, AML12 cells were exposed to 30 mM APAP for 12 h to establish an AILI model and subjected to the indicated treatments for each group. Cell viability, apoptosis, proliferation, and autophagy-related markers were evaluated using the CCK-8 assay, immunohistochemistry, immunofluorescence, and Western blotting. In vivo, an APAP-induced liver injury model was established in C57BL/6J mice, followed by the indicated treatments for each group. Liver injury, regeneration and autophagy were assessed by serum biochemical analysis, histopathological examination, ELISA, qPCR, Western blotting, and transmission electron microscopy (TEM). Mechanistically, the involvement of PINK1/Parkin-mediated mitophagy in silibinin-induced hepatoprotection was systematically investigated. We created the figure with “BioRender.com.”

We used the AML12 cell line (mouse normal hepatocyte cell line) and C57BL/6J mice as study models. Cells and animals were subjected to APAP to induce hepatocellular damage. Silibinin was used to treat hepatocellular damage. An autophagy inhibitor, here PINK1-siRNA for the in vitro experiment and 3-Methyladenine (3-MA) for the in vivo experiment, was used examining whether the beneficial effect of silibinin was mediated by upregulating mitophagy (Fig. 1).

#### In vitro experiments

**Fig. 2:**
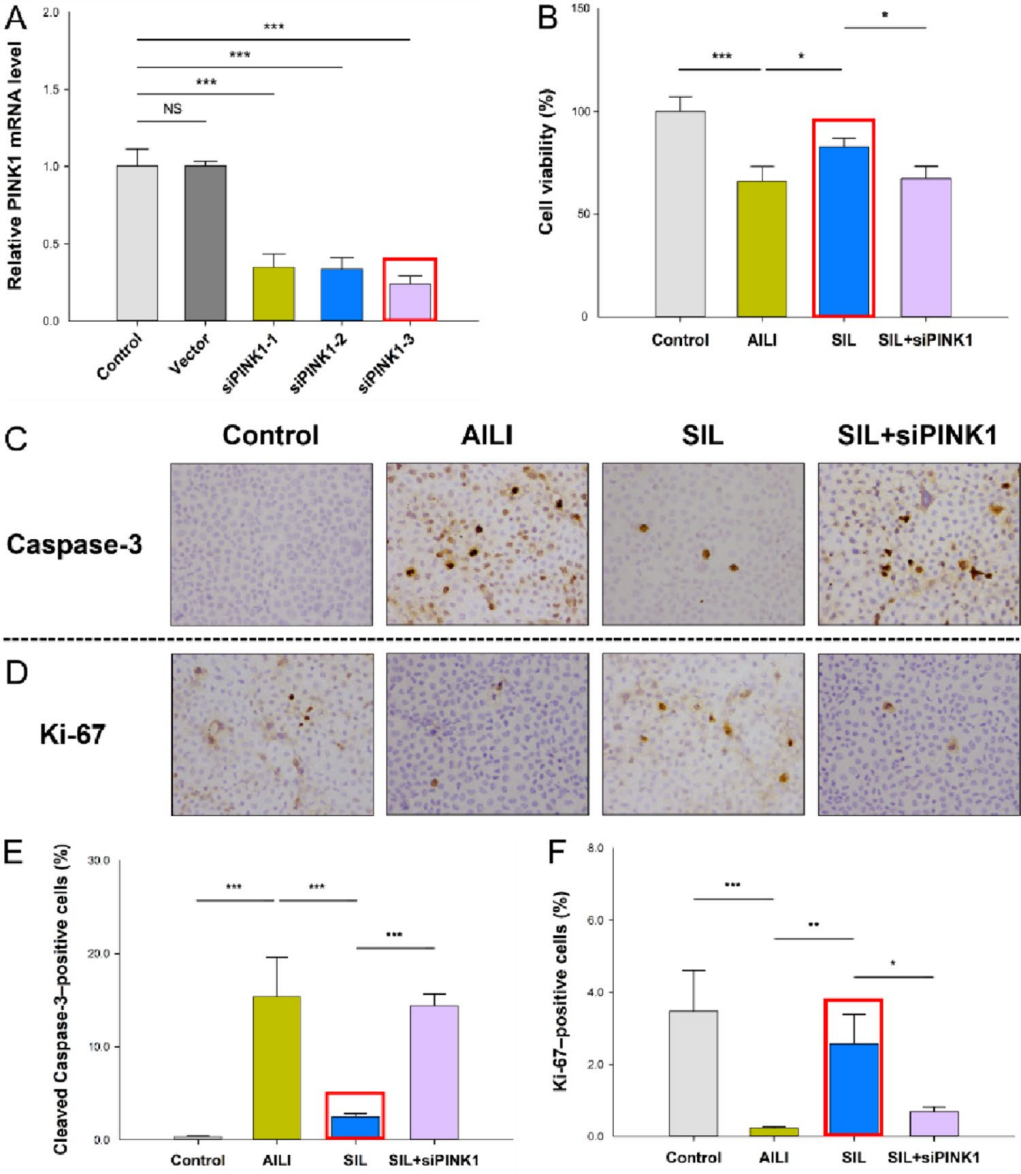
Silibinin attenuates acetaminophen-induced injury in AML12 cells by reducing apoptosis and promoting cell proliferation. (**A**) Validation of PINK1 knockdown efficiency in AML12 cells by qPCR; (**B**) Cell viability analysis; (**C**) Immunohistochemical staining of cleaved caspase-3 in AML12 cells; (**D**) Immunohistochemical staining of Ki-67 in AML12 cells; (**E**) Quantification of cleaved caspase-3 immunohistochemical staining in AML12 cells; (**F**) Quantification of Ki-67 immunohistochemical staining in AML12 cells. *P < 0.05, **P < 0.01, ***P < 0.001

AML12 cells were obtained from Procell system Co., Ltd. (#CL-0602). Complete DMEM/F12 medium was prepared by supplementing the DMEM/F12 medium (Procell system Co., Ltd., # PM150312) with 10% fetal bovine serum (FBS), 0.5% Insulin-Transferrin-Selenium, 40 ng/mL Dexamethasone, and 1% penicillin–streptomycin. All cells were cultured in a humidified incubator at 37 °C with 5% CO₂. PINK1 siRNAs (three independent siRNAs designated siPINK1-1, siPINK1-2, and siPINK1-3) were synthesized by Beijing Tsingke Biotech Co., Ltd. As shown in Fig. 2A, qPCR analysis demonstrated that siPINK1-3 exhibited the highest knockdown efficiency among the three siRNAs. Therefore, siPINK1-3 was selected for subsequent transfection experiments. Based on our preliminary dose-response experiments in AML12 cells, 30 mM APAP was selected to establish the AILI model because it produced moderate and reproducible hepatocellular injury while maintaining sufficient viable cells for downstream mechanistic studies.

The cell culture experiments were divided into four groups:

(1) Control group: Since both drugs are dissolved in Dimethyl sulfoxide (DMSO), cells were first treated with complete culture medium containing DMSO at a volume equal to that of the APAP solvent for 12 h, followed by treatment with complete culture medium containing DMSO at a volume equal to that of the SIL solvent for 24 h;

(2) AILI model group: Cells were treated with complete culture medium containing 30 mM APAP for 12 h, followed by treatment with complete culture medium containing DMSO at a volume equal to that of the SIL solvent for 24 h;

(3) SIL group: Cells were treated with complete culture medium containing 30 mM APAP for 12 h, followed by treatment with complete culture medium containing 50 µM SIL for 24 h;

(4) SIL + PINK1-siRNA group: Cells were treated with PINK1-siRNA for 24 h, then cells were treated with complete culture medium containing 30 mM APAP for 12 h, followed by treatment with complete culture medium containing 50 µM SIL for 24 h.

Experiments were independently repeated three times, and cell samples were collected immediately after the experiments for further analysis. We aimed to elucidate the role of SIL in modulating autophagy, hepatocyte proliferation, and AILI, with a particular focus on the involvement of PINK1/Parkin–mediated mitophagy and its relationship with hepatocyte proliferation and AILI.

#### In vivo experiments

40 male C57BL/6J mice (6–8 weeks old, SPF grade) were purchased from SPF (Beijing) Biotechnology Co., Ltd. The mice were housed under controlled conditions with a temperature of 25 °C, humidity of 60%, and a 12-hour light/dark cycle. Drinking water, feed, bedding, and other supplies were sterilized by autoclaving, and the cages were cleaned weekly. All animal procedures were conducted in accordance with the requirements of the Animal Ethics Committee of Kangtai Medical Testing Service Hebei Co., Ltd. (Ethics Approval No. MDL2025-04-23-01).

After one week of acclimatization, the 40 mice were randomly divided into four groups, with 10 mice in each group:

The AILI mouse model was established by intraperitoneal injection of 300 mg/kg (body weight) APAP, with reference to the method of Mossanen et al. [26]. The SIL stock solution was first prepared and then thoroughly mixed with normal saline (NS) to obtain a uniform gavage suspension.

(1) Control group (*n* = 10): Mice were intraperitoneally injected with DMSO at a volume equal to that of the APAP solvent and observed for 12 h, followed by intragastric administration of an equal volume of NS corresponding to SIL once per day for 5 days;

(2) AILI model group (*n* = 10): Mice were intraperitoneally injected with 300 mg/kg (body weight) APAP and observed for 12 h, followed by intragastric administration of NS corresponding to SIL once per day for 5 days;

(3) SIL group (*n* = 10): Mice were intraperitoneally injected with 300 mg/kg (body weight) APAP and observed for 12 h, followed by intragastric administration of SIL (100 mg/kg) once per day for 5 days;

(4) SIL + 3-MA group (*n* = 10): Mice were intraperitoneally injected with 300 mg/kg (body weight) APAP and observed for 12 h, followed by intragastric administration of SIL (100 mg/kg) and intraperitoneal injection of 3-MA solution (10 mg/kg) once per day for 5 days.

Mice were not subjected to fasting before treatment, as the intervention was intended to be administered under normal physiological conditions that reflect typical feeding behavior. After the final dose, mice were fasted for approximately 12 h prior to sacrifice to minimize variability in serum biochemical parameters and liver histology related to recent food intake, thereby ensuring greater accuracy and consistency of the results. Liver tissue and blood samples were collected for subsequent analysis.

### Materials

Chemicals and solutions were mainly purchased from Sigma-Aldrich, MedChemExpress and Procell system. E.g., Silibinin (Sigma-Aldrich, #S0417), Acetaminophen (MedChemExpress, #HY-66005), 3-Methyladenine (MedChemExpress, #HY-19312), and FBS (Procell system, #164210).

### Cell counting kit-8 (CCK-8) assay

AML12 cells (1 × 10^4^ cells/well) were seeded in a 96-well plate and incubated for 24 h to allow attachment. Treatments were applied according to the experimental design. Subsequently, 10 µL of CCK-8 solution was added to each well. After 2 h of incubation at 37 °C, absorbance was measured at 450 nm using a microplate reader.

### Liver function assessment

Blood samples were collected and centrifuged at 3,500 rpm for 15 min at 4 °C to obtain serum. The prepared serum was then analyzed using an automatic biochemical analyzer (Xinrui XR220Plus, China) to determine liver function related parameters, including alanine aminotransferase (ALT), aspartate aminotransferase (AST), total bilirubin (TBIL), and albumin (ALB).

### Hematoxylin–Eosin (H&E) staining

Mouse livers were rapidly excised and fixed in 4% paraformaldehyde (Beyotime, #P0099) for 24 h. The fixed liver tissues were then dehydrated through a graded ethanol series, cleared in xylene, and embedded in paraffin. Paraffin-embedded tissues were sectioned at 4 μm thickness using a microtome (Leica RM2235, Germany) and mounted onto glass slides.

The paraffin sections were deparaffinized in xylene and rehydrated through a graded ethanol series to distilled water. Sections were stained with hematoxylin for approximately 1 min, rinsed under running tap water, and blued in an ammonia solution. Subsequently, sections were counterstained with eosin for a few seconds, rinsed under running water, dehydrated through a graded ethanol series, cleared in xylene, and mounted with a resinous mounting medium (ZSGB-BIO, #ZLI-9555). Necrotic areas were quantitatively analyzed using QuPath (version 0.6.0). Whole-slide images of the tissue sections were obtained using a whole slide scanner (WISLEAP WS-10, China) and subsequently imported into the analysis software for identification of necrotic regions based on morphological criteria. The percentage of necrotic area relative to the total tissue area was calculated.

### ELISA assay

The levels of IL-6 and Cyclin D1 in liver tissues were determined using commercial ELISA kits (IL-6: MDL, #MD123475; Cyclin D1: MDL, #MD26682) according to the instructions of the manufacturer.

### SOD and MDA assay

Hepatic superoxide dismutase (SOD) and Malondialdehyde (MDA) levels in liver tissues were measured using commercial assay kits (SOD, Nanjing Jiancheng Bioengineering Institute, #A001-3; MDA, Nanjing Jiancheng Bioengineering Institute, #A003-1), following the instructions provided with the kits.

### Immunohistochemistry

Samples were fixed with 4% paraformaldehyde, followed by permeabilization with 0.2% Triton X-100 when required. After three washes with PBS (pH 7.4), slides were blocked with goat serum at 37 °C for 30 min. Samples were then incubated with primary antibodies (Ki-67: Affinity, #AF0198; cleaved caspase-3: Affinity, #AF7022) overnight at 4 °C, followed by three PBS washes. Appropriate species-specific secondary antibodies were applied for 1 h at 37 °C and washed three times with PBS. Signals were developed using DAB solution (ZSGB-BIO, #ZLI-9017) and observed under a microscope. Finally, slides were counterstained with hematoxylin, dehydrated through a graded ethanol series, cleared in xylene, and mounted with a resinous mounting medium (ZSGB-BIO, #ZLI-9555). Slides were digitized using a slide scanner (WISLEAP WS-10, China). Immunohistochemical results were quantitatively analyzed using QuPath (version 0.6.0) and AIpathwell (version 2.3.1). For in vitro experiments, each group contained three samples. For in vivo experiments, each group contained ten samples. Positive cells were identified using the built-in cell detection algorithm.

### Immunofluorescence

AML12 cells were fixed with 4% paraformaldehyde for 20 min, permeabilized with 0.2% Triton X-100 for 20 min, and washed three times with PBS (pH 7.4). Cells were blocked with goat serum at 37 °C for 30 min, followed by incubation with primary antibody (LC3: Proteintech, #14600-1-AP) overnight at 4 °C. After three PBS washes, cells were incubated with species-specific fluorescent secondary antibodies at 37 °C for 1 h. Nuclei were stained with DAPI (Yeasen Biotechnology Co., Ltd, #40728ES03) for 10 min at room temperature in the dark. After three final washes with PBS, slides were mounted with anti-fade medium and stored at 4 °C in the dark. Each group contained three representative merged immunofluorescence images, resulting in a total of 12 merged images across the four groups. Quantitative analysis of LC3 immunofluorescence was performed by measuring the mean fluorescence intensity using ImageJ software (version 1.51j8, USA). During analysis, identical threshold settings (Min: 61, Max: 255) were uniformly applied to all images to ensure consistency and minimize analytical bias.

### Quantitative PCR

Frozen liver tissues were ground into a fine powder, and total RNA was extracted using TRIpure reagent (Aidlab Biotechnologies, #RN0102). cDNA was synthesized using a reverse transcription kit (EXONGEN, #A502) according to the instructions of the manufacturer. Quantitative PCR was then performed on a LongGene Q2000B real-time PCR system (LongGene, China). Primer sequences are listed in Table 1. The primers were synthesized by Beijing Tsingke Biotech Co., Ltd. Relative mRNA expression levels of target genes were calculated using the 2^-ΔΔCt method, with GAPDH serving as the internal control.

**Table 1:** qPCR primer information

Gene	Forward primer (5′–3′)	Reverse primer (5′–3′)
PINK1	CGACAACATCCTTGTGGAGTGG	CATTGCCACCACGCTCTACACT
Parkin	TCTTCCAGTGTAACCACCGTC	GGCAGGGAGTAGCCAAGTT
PRKAA1	ACCTGAGAACGTCCTGCTTG	GGCCTGCGTACAATCTTCCT
LC3B	CACTGCTCTGTCTTGTGTAGGTTG	TCGTTGTGCCTTTATTAGTGCATC
p62	ATGGTGCACCCCAATGTGAT	CTGCACAGGTCGTAGTCTGG
GAPDH	AGGTCGGTGTGAACGGATTTG	TGTAGACCATGTAGTTGAGGTCA

### Western blotting

Hepatic samples were homogenized in an appropriate volume of RIPA lysis buffer (Beyotime, #P0013) supplemented with protease inhibitor (MDL, #MD912893) and phosphatase inhibitor (TargetMol, #C0002) to ensure complete sample lysis and preservation of protein phosphorylation. After lysis, the samples were centrifuged to remove debris, and the supernatant was collected as total protein. Protein concentration was determined using the BCA assay, and the protein samples were adjusted to a final concentration of 4 mg/mL for subsequent Western blot analysis. Total protein was mixed with 5× SDS-PAGE loading buffer. Samples were denatured at 95 °C for 10 min and placed on ice. The Nitrocellulose (NC) membrane was immersed in methanol for 2–3 min. Proteins were separated by SDS-PAGE and transferred onto the NC membrane. The membrane was blocked with blocking solution (Servicebio, #G2052) at room temperature for 1 h on a shaker. Primary antibodies (PINK1: Proteintech, #23274-1-AP, dilution ratio: 1:1000; Parkin: Proteintech, #14060-1-AP, 1:1000; AMPK: Proteintech, #10929-2-AP, 1:2000; Phospho-AMPK alpha (Thr172): Cell Signaling Technology, #2531, 1:500; LC3: Proteintech, #14600-1-AP, 1:2000; p62: Proteintech, #31403-1-AP, 1:1000; GAPDH: Proteintech, #60004-1, 1:10000) were diluted to the appropriate concentrations and incubated with the membrane overnight at 4 °C. After washing the membrane three times with 1× TBST for 10 min each, the membrane was incubated with the secondary antibody at room temperature in the dark for 1 h with gentle shaking. Following three washes with 1× TBST, fresh Enhanced chemiluminescence (ECL, HaiGene, #M2301) working solution was applied to the membrane and allowed to react for 1–2 min at room temperature. Chemiluminescent signals were captured using a chemiluminescence imaging system (CLINX ChemiScope 6100, China), and quantitative analysis was performed using ImageJ software (version 1.51j8, USA).

### Transmission electron microscopy (TEM) analysis

Fresh liver tissues were cut into 1 mm³ blocks and fixed in 2.5% glutaraldehyde at 4 °C for about 4 h. After three washes in 0.1 M phosphate buffer, samples were post-fixed in 1% osmium tetroxide at 4 °C for 4 h. Tissues were dehydrated through a graded ethanol series (30%–100%), followed by propylene oxide, and then infiltrated with epoxy resin using gradual epoxy resin–propylene oxide mixtures. Samples were embedded in resin and polymerized at 60 °C for 48 h.

Semi-thin sections were prepared for orientation, and 70 nm ultrathin sections were cut using an ultramicrotome (Leica UC7, Germany) and collected on copper grids. Sections were stained with uranyl acetate and lead citrate, followed by thorough rinsing. Ultrastructural morphology was examined under a transmission electron microscope (JEOL JEM-1400, Japan), and representative images were captured for analysis. For TEM ultrastructure quantification, five randomly selected fields were analyzed in a blinded manner at identical magnifications (scale bar = 500 nm). The percentage of damaged mitochondria and the number of autolysosomes were quantified using ImageJ software (version 1.51j8, USA). Damaged mitochondria were identified based on morphological features including swelling, cristae disruption, vacuolization, or membrane rupture. Autolysosomes were defined as single-membrane vesicular structures containing partially degraded cytoplasmic components or organelles.

### Statistical analysis

Data analysis was performed with the statistical software SPSS 27.0 and SigmaPlot 13.0. Differences between groups were assessed using one-way ANOVA followed by LSD post hoc test when the data were normally distributed or approximately normally distributed with homogeneity of variance. For data that were normally distributed or approximately normally distributed but with unequal variances, Welch’s ANOVA followed by Tamhane’s T2 post hoc test was applied. For data that did not follow a normal distribution, the Kruskal-Wallis test was used. Statistical significance was defined as *P* < 0.05.

## Results

### Silibinin significantly restores hepatocyte viability in vitro and attenuates APAP-induced body weight loss in vivo

In the in vitro study, the CCK-8 assay showed a significant reduction in AML12 cell viability in the AILI model group compared with the control group, while SIL treatment markedly restored cell viability (Fig. 2B). Consistently, immunohistochemical analysis revealed a significant increase in cleaved caspase-3 expression in the AILI model group, and SIL treatment significantly attenuated its expression (Fig. 2C, E). These findings suggest that SIL exerts a protective effect against AILI-induced hepatocyte apoptosis.

**Fig. 3:**
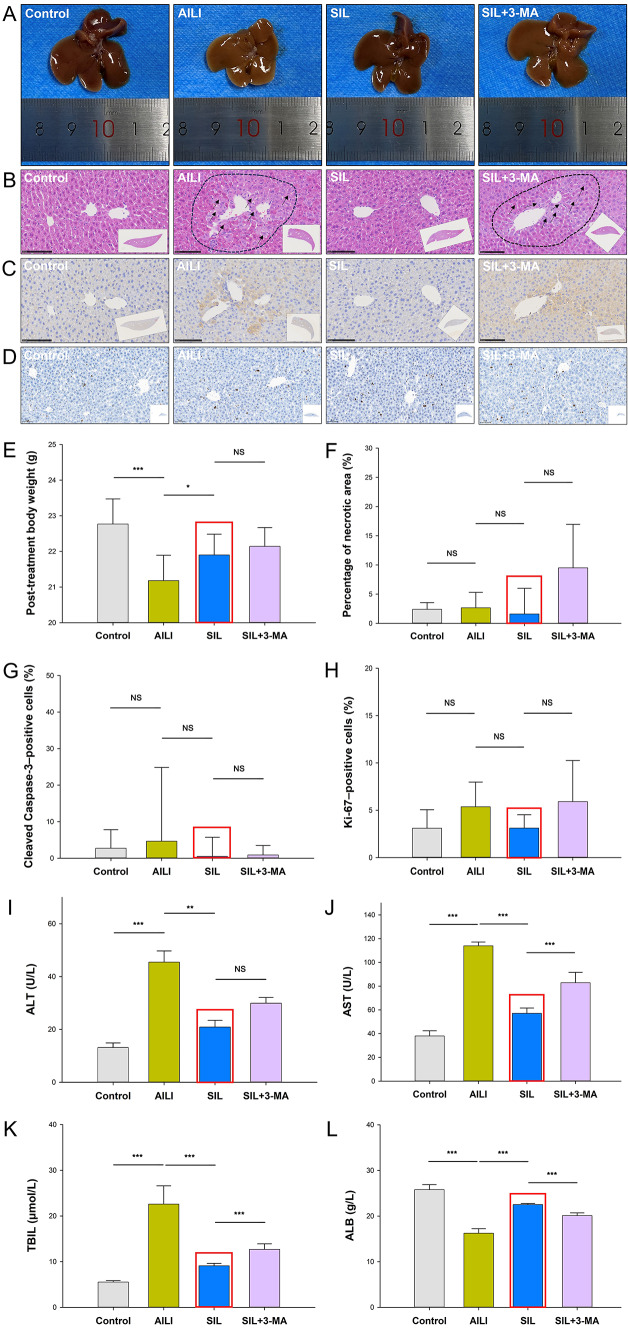
Silibinin alleviates acetaminophen-induced liver dysfunction in C57BL/6J mice. (**A**) Representative images of explanted mouse livers; (**B**) H&E staining results; (**C**) Cleaved caspase-3 immunohistochemical staining results; (**D**) Ki-67 immunohistochemical staining results; (**E**) Body weight of mice after intervention; (**F**) Quantification of the percentage of necrotic area in H&E-stained sections; (**G**) Quantification of cleaved caspase-3 immunohistochemical staining; (**H**) Quantification of Ki-67 immunohistochemical staining; (**I**) Serum ALT levels; (**J**) Serum AST levels; (**K**) Serum TBIL levels; (**L**) Serum ALB levels

In the in vivo study, body weight analysis was performed for each group of mice. Before the start of the experiment, no significant differences in body weight were observed among the groups. At the end of the experiment, mice in the AILI model group exhibited a significant reduction in body weight (6.98%) compared with the control group, while SIL treatment significantly alleviated this weight loss (3.40%) (Fig. 3E), indicating that SIL attenuates APAP-induced body weight loss.

### Silibinin effectively alleviated AILI-induced liver dysfunction in mice

The H&E and cleaved caspase-3 immunohistochemical staining showed that the liver structure of mice in the control group was normal, with well-organized hepatic cords. In contrast, hepatocytes in the AILI model group exhibited increased necrosis and apoptosis observed in portions of both the central vein and portal vein areas compared to the control group. However, these differences did not reach statistical significance. Compared with the AILI model group, the SIL-treated group showed a tendency toward histopathological improvement, with partially reduced necrosis and apoptosis, suggesting that SIL may alleviate AILI. However, these differences did not reach statistical significance. In contrast, liver injury appeared to be aggravated in the SIL + 3-MA group, implying that the autophagy inhibitor 3-MA may partially attenuated the potential hepatoprotective effect of SIL (Fig. 3B, C, F, G).

Consistently, serum biochemical analysis showed that compared with the control group, mice in the AILI model group exhibited significantly increased levels of ALT, AST, and TBIL, along with a marked decrease in ALB, suggesting liver dysfunction. In contrast, treatment with SIL significantly reduced ALT, AST, and TBIL levels while markedly elevating ALB levels, suggesting a recovery of liver function (Fig. 3I, J, K, L). However, the degree of improvement was attenuated in the SIL + 3-MA group, implying that inhibition of autophagy weakened the hepatoprotective effect of SIL.

**Fig. 4:**
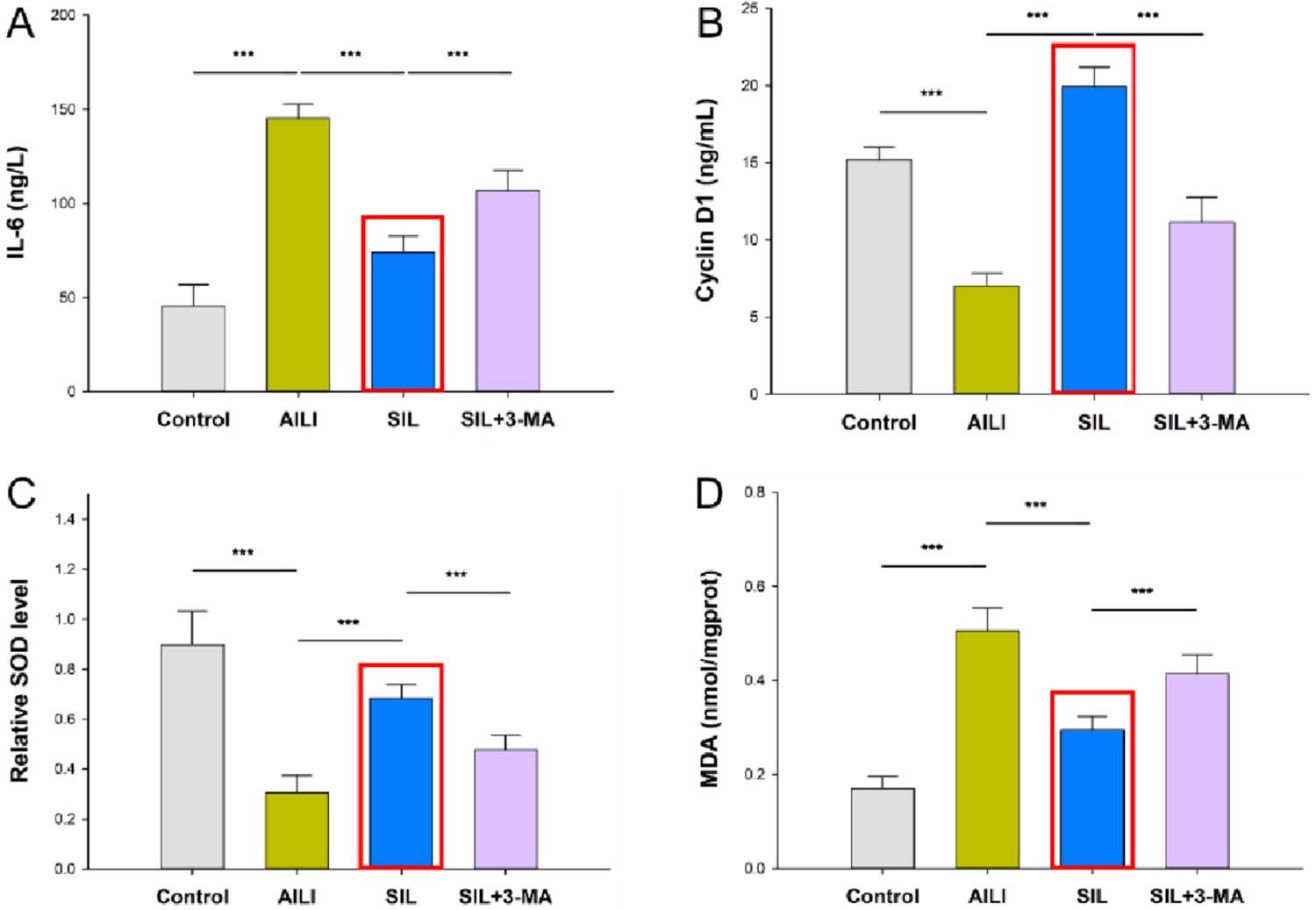
Silibinin attenuates acetaminophen-induced hepatic inflammation and mitochondrial injury, while enhancing antioxidant capacity and promoting hepatocyte proliferation in C57BL/6J mice. (**A**) IL-6 levels in mouse liver; (**B**) Cyclin D1 levels in mouse liver; (**C**) SOD levels in mouse liver; (**D**) MDA levels in mouse liver

Furthermore, ELISA results demonstrated that compared with the control mice, hepatic IL-6 levels were significantly elevated in the AILI model group, suggesting exacerbation of hepatic inflammation. Compared with the AILI model group, hepatic IL-6 levels were markedly decreased in the SIL-treated mice, suggesting that SIL effectively attenuated liver inflammation. However, hepatic IL-6 levels increased again in the SIL + 3-MA group (Fig. 4A), suggesting that inhibition of autophagy partially diminished the anti-inflammatory effect of SIL.

### Silibinin markedly promotes hepatocyte proliferation following AILI

In the in vitro study, immunohistochemical analysis showed that Ki-67 expression was significantly decreased in AML12 cells in the AILI model group compared with the control group. In contrast, SIL treatment significantly increased Ki-67 expression (Fig. 2D, F). Moreover, the CCK-8 assay demonstrated increased cell viability in SIL-treated cells, which was consistent with enhanced hepatocyte proliferation (Fig. 2B).

Similarly, in the in vivo study, ELISA results revealed a significant reduction in Cyclin D1 levels in mice from the AILI model group relative to the control group, suggesting suppressed hepatocyte proliferation. Treatment with SIL substantially elevated Cyclin D1 levels compared with the AILI model group, indicating that SIL promoted hepatocyte proliferation. Notably, this increase in Cyclin D1 was attenuated in the SIL + 3-MA group (Fig. 4B), implying that autophagy inhibition weakened the pro-proliferative effect of SIL.

Interestingly, Cyclin D1 levels and hepatic Ki-67-positive cell rates in mice exhibited partially divergent trends among the groups, although the differences in Ki-67 staining did not reach statistical significance (Figs. 3H and 4B). Cyclin D1, a key regulator of the G1/S transition, primarily reflects cell cycle initiation, whereas Ki-67 serves as a marker of actively proliferating cells. Considering that these observations were obtained after 5 days of different treatment conditions, the elevated Cyclin D1 levels observed in the SIL-treated group suggest enhanced cell cycle readiness and controlled liver regeneration, indicating that hepatic repair may have progressed to a certain extent. In contrast, the relatively increased Ki-67 positivity observed in the AILI and SIL + 3-MA groups may reflect compensatory or dysregulated proliferation in response to aggravated liver injury, particularly under conditions of autophagy inhibition. Taken together, these findings suggest that silibinin promotes orderly hepatocyte regeneration, while autophagy inhibition may lead to potentially maladaptive proliferative responses.

### Silibinin significantly improved hepatocyte mitochondrial function

The SOD assay results demonstrated that hepatic SOD activity was significantly reduced in livers from mice of the AILI model group compared with the control group, reflecting a decline in hepatic antioxidant capacity. SIL treatment markedly increased SOD activity, indicating an augmentation of hepatic antioxidant capacity. In contrast, SOD activity was significantly reduced in the SIL + 3-MA group (Fig. 4C).

Complementary to this finding, MDA assay results demonstrated that hepatic MDA levels were significantly elevated in livers from mice of the AILI model group compared with the control group, reflecting increased lipid peroxidation and oxidative stress. SIL treatment markedly reduced MDA levels, indicating alleviation of oxidative damage. In contrast, MDA levels in the SIL + 3-MA group rose (Fig. 4D), suggesting that autophagy inhibition weakened the antioxidant effect of SIL.

### Silibinin effectively restored AILI-induced suppression of hepatic autophagy

**Fig. 5:**
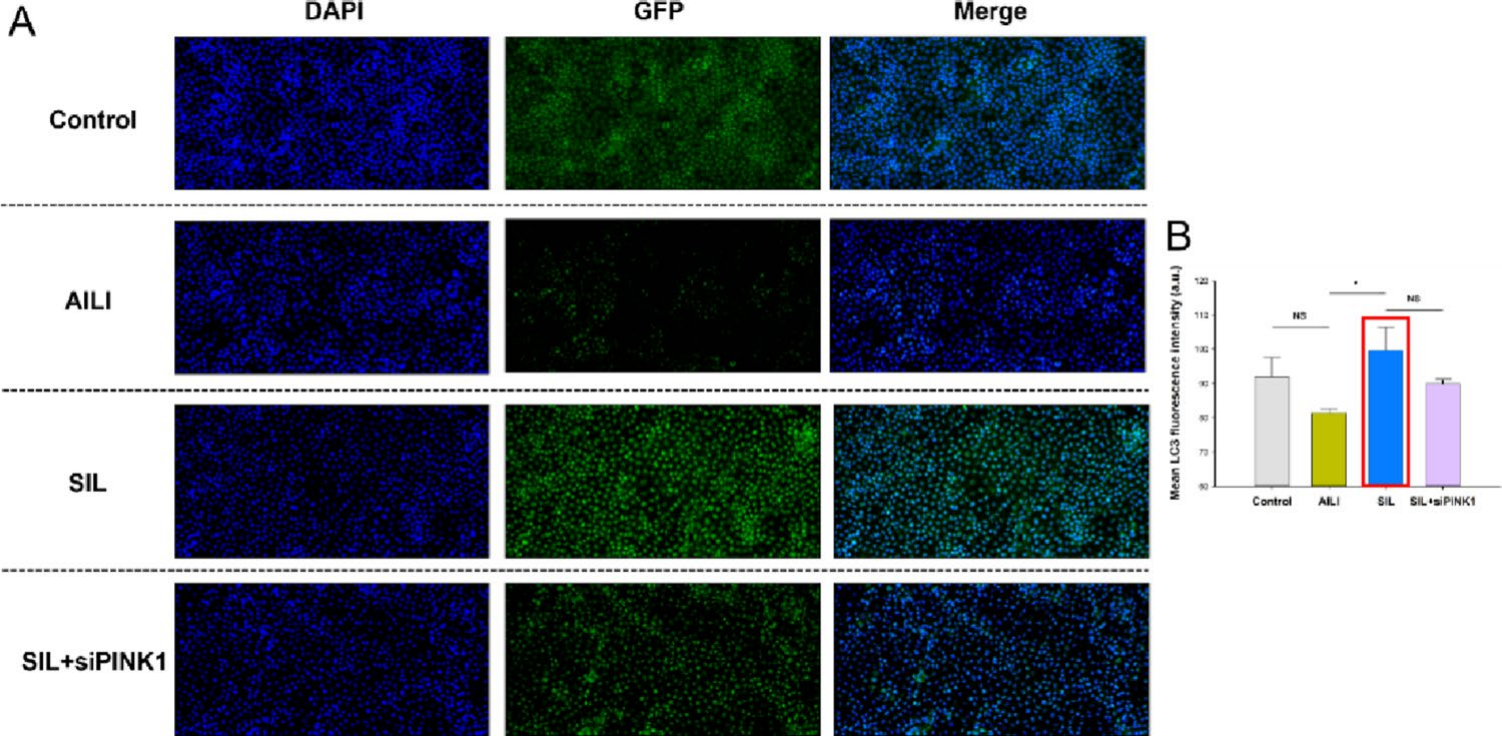
Immunofluorescence staining shows that silibinin restores LC3 expression suppressed by AILI in AML12 cells. (**A**) Representative images of LC3 immunofluorescence staining in each group. (**B**) Quantification of LC3 immunofluorescence in AML12 cells

In the in vitro study, immunofluorescence analysis showed that LC3 expression in AML12 cells was decreased in the AILI model group compared with the control group, although the difference did not reach statistical significance. In contrast, LC3 expression was significantly increased in the SIL-treated group (Fig. 5A, B), suggesting that SIL improved AILI-induced autophagy inhibition in AML12 cells.

**Fig. 6:**
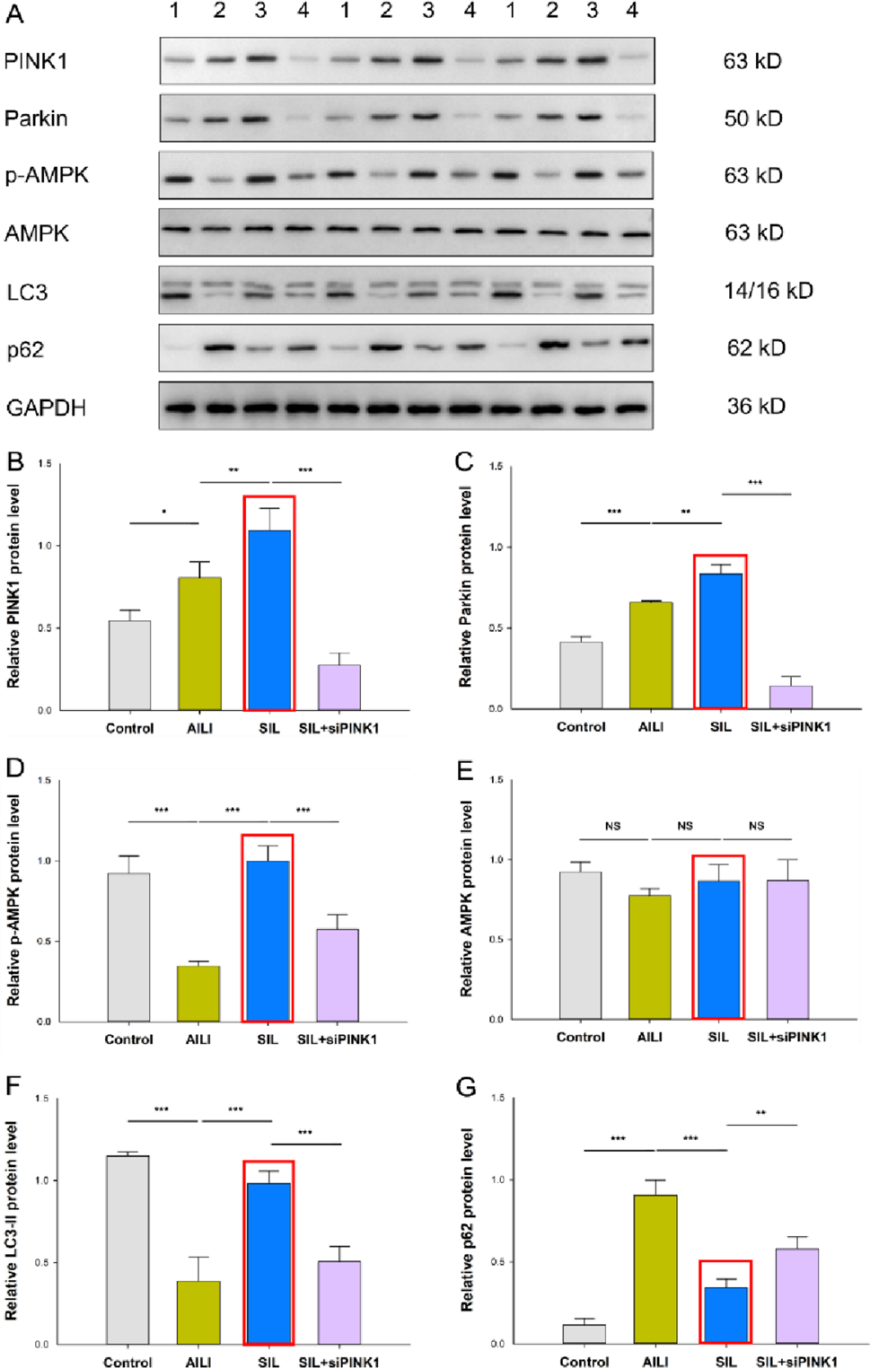
The Western blot results demonstrate that silibinin alleviates AILI-induced autophagy inhibition at the protein level in AML12 cells. (**A**) Representative Western blot analysis of PINK1, Parkin, phosphorylated AMPK (p-AMPK), total AMPK, LC3, and p62 protein expression in different experimental groups; (**B**) PINK1 protein levels; (**C**) Parkin protein levels; (**D**) Phosphorylated AMPK (p-AMPK) protein levels; (**E**) Total AMPK protein levels; (**F**) LC3-II protein levels; (**G**) p62 protein levels. Abbreviations: (1) Control group; (2) AILI model group; (3) SIL group, silibinin treatment group; (4) SIL+PINK1-siRNA group

Consistently, Western blot analysis revealed that relative to the control, AML12 cells in the AILI model group exhibited reduced Phospho-AMPK alpha (Thr172) and LC3-II levels and elevated p62 expression, suggesting suppression of autophagy. In contrast, SIL treatment significantly increased Phospho-AMPK alpha (Thr172) and LC3-II expression and decreased p62 levels compared with the AILI model group (Fig. 6A, D, F, G), indicating partial restoration of autophagy.

**Fig. 7:**
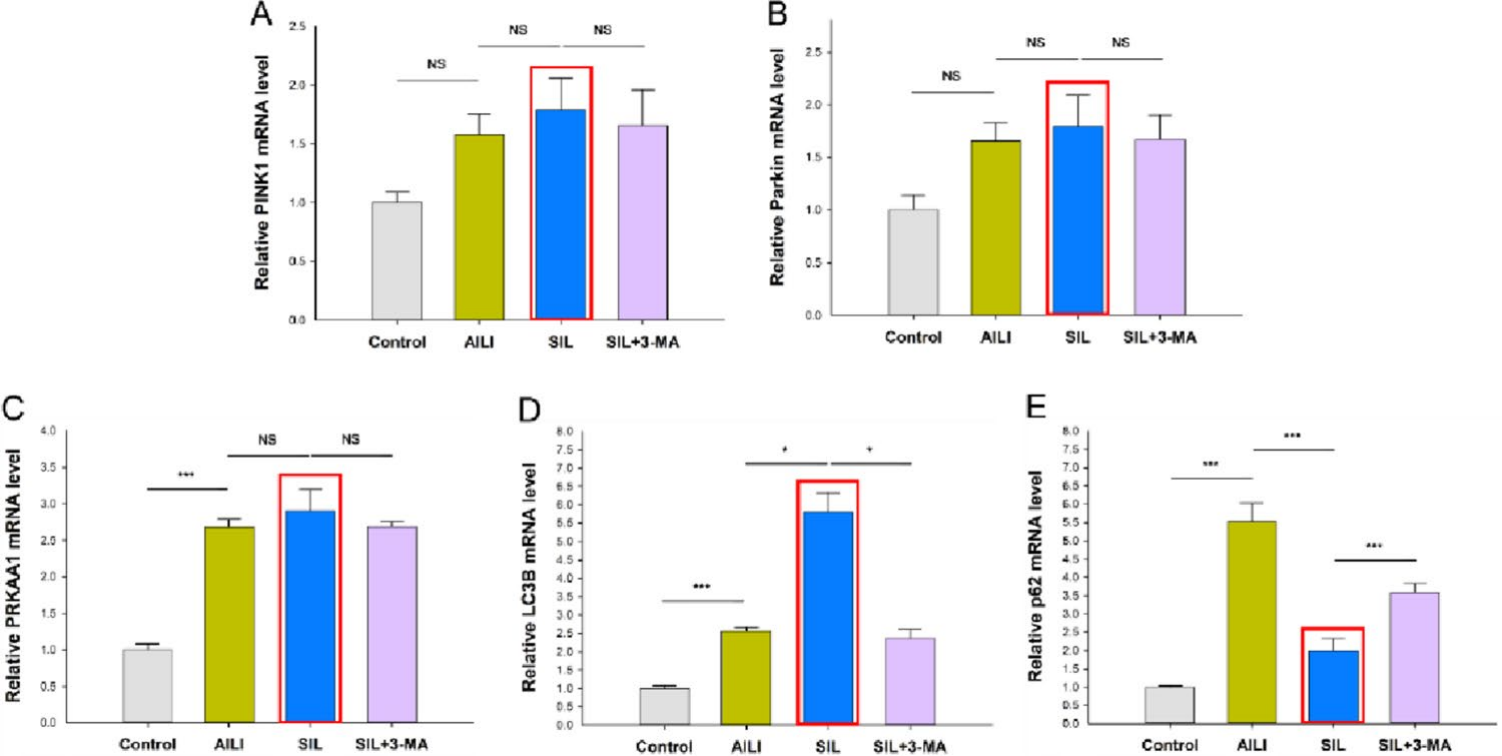
The qPCR results show that silibinin alleviates AILI-induced autophagy inhibition at the transcriptional level in the liver of C57BL/6J mice. (**A**) PINK1 mRNA levels in mouse liver; (**B**) Parkin mRNA levels in mouse liver; (**C**) PRKAA1 mRNA levels in mouse liver; (**D**) LC3B mRNA levels in mouse liver; (**E**) p62 mRNA levels in mouse liver

In the in vivo study, qPCR analysis showed that hepatic mRNA levels of PRKAA1, LC3, and p62 were elevated in mice of the AILI model group compared with control mice, reflecting impaired autophagy. SIL treatment further increased LC3 expression while decreasing p62 levels relative to the AILI model group, suggesting autophagy activation. By contrast, the SIL + 3-MA group exhibited a marked reduction in LC3 expression and a significant increase in p62 levels compared with the SIL group (Fig. 7C, D, E), suggesting autophagy inhibition.

**Fig. 8:**
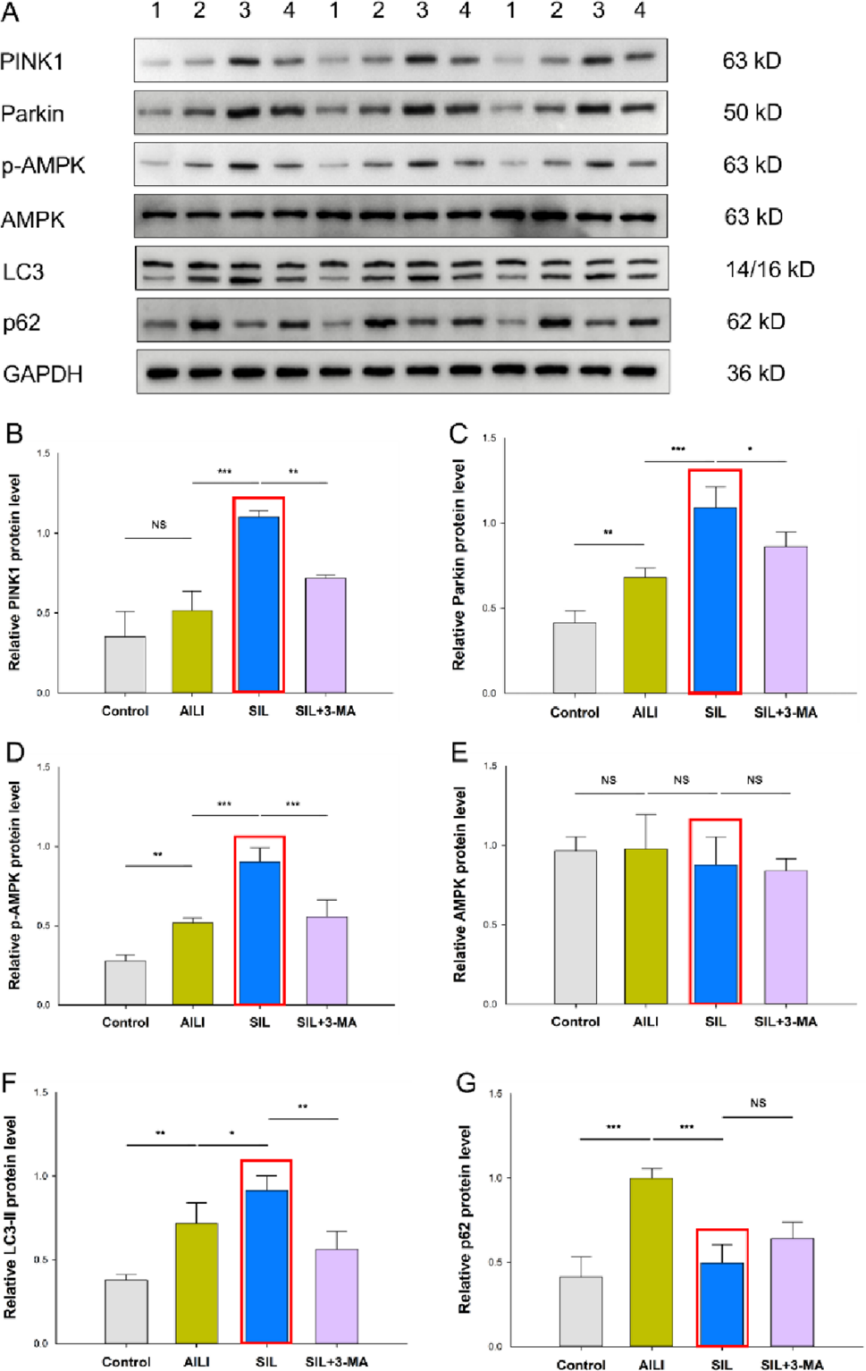
The Western blot results demonstrate that silibinin attenuates AILI-induced autophagy inhibition at the protein level in the liver of C57BL/6J mice. (**A**) Representative Western blot analysis of PINK1, Parkin, phosphorylated AMPK (p-AMPK), total AMPK, LC3, and p62 protein expression in different experimental groups; (**B**) PINK1 protein levels; (**C**) Parkin protein levels; (**D**) Phosphorylated AMPK (p-AMPK) protein levels; (**E**) Total AMPK protein levels; (**F**) LC3-II protein levels; (**G**) p62 protein levels. Abbreviations: (1) Control group; (2) AILI model group; (3) SIL group, silibinin treatment group; (4) SIL + 3-MA group

Similarly, Western blot analysis of hepatic tissues revealed that Phospho-AMPKα (Thr172), LC3-II, and p62 levels were increased in the AILI model group compared with controls, suggesting a blockade of autophagic flux. SIL treatment further elevated Phospho-AMPKα (Thr172), LC3-II levels and reduced p62 expression, suggesting activation of autophagy. In comparison, autophagy appeared to be suppressed in the SIL + 3-MA group relative to the SIL group (Fig. 8A, D, F, G).

### Silibinin alleviated AILI by activating mitophagy via the PINK1/Parkin pathway

In the in vitro study, Western blot analysis showed that PINK1 and Parkin levels were markedly elevated in AML12 cells of the AILI model group compared with the control group, suggesting that AILI-induced mitochondrial damage likely triggered stress-induced mitophagy (Fig. 6A, B, C). However, decreased Phospho-AMPKα (Thr172) and LC3-II, along with increased p62, indicated that mitophagy was not effectively completed. SIL treatment upregulated PINK1, Parkin and LC3-II while decreasing p62, indicating effective activation of mitophagy. Notably, compared with the SIL-treated group, AML12 cells treated with SIL+PINK1-siRNA exhibited substantially reduced PINK1 and Parkin expression, decreased LC3-II, and increased p62 (Fig. 6A, B, C, D, F, G), indicating mitophagy inhibition.

In the in vivo study, qPCR analysis showed that, compared with the AILI model group, PINK1 and Parkin expression in SIL-treated mice were slightly elevated, although the differences did not reach statistical significance (Fig. 7A, B). LC3 expression was significantly upregulated and p62 expression were significantly downregulated, suggesting activation of autophagy. In the SIL + 3-MA group, LC3 expression was decreased and p62 expression was increased compared with the SIL group (Fig. 7D, E), implying inhibition of SIL-induced autophagy.

Similarly, Western blot analysis demonstrated that, compared with the AILI model group, the protein levels of PINK1, Parkin, and LC3-II were significantly upregulated and p62 was significantly downregulated in the SIL group (Fig. 8A, B, C, F, G), suggesting activation of mitophagy. Mitophagy appeared to be partially inhibited in the SIL + 3-MA group relative to the SIL group (Fig. 8A, B, C, D, F, G).

**Fig. 9:**
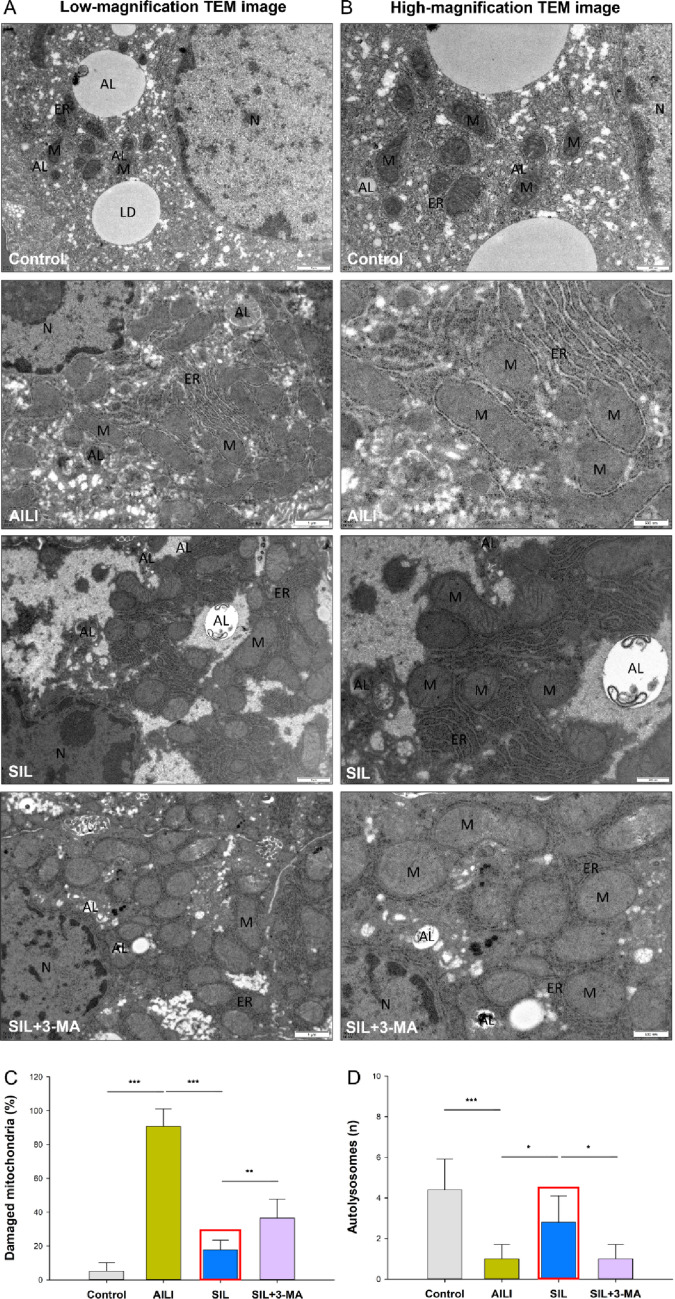
TEM analysis revealed that silibinin activates mitophagy and attenuates mitochondrial injury in hepatocytes of C57BL/6J mice. (**A**) Representative low-magnification TEM images; (**B**) Representative high-magnification TEM images; (**C**) Quantification of the percentage of damaged mitochondria; (**D**) Quantification of the number of autolysosomes per field. Abbreviations: Autolysosome, AL; Endoplasmic reticulum, ER; Lipid droplet, LD; Mitochondrion, M; Nucleus, N

Furthermore, transmission electron microscopy (TEM) was employed to examine the ultrastructural differences in hepatocytes among the mouse groups. In the control group, hepatocytes exhibited a generally regular morphology, with oval-shaped nuclei. Mitochondria in the cytoplasm were rod-shaped or oval, with intact structures and clearly visible cristae. The endoplasmic reticulum displayed a well-defined structure, and a number of lipid droplets and autolysosomes were observed in the cytoplasm. In the AILI model group, hepatocytes showed irregular morphology with distorted nuclei. Although most mitochondria appeared structurally intact, their cristae were markedly blurred. The endoplasmic reticulum structure remained relatively clear, while only a few autolysosomes were detected. In the SIL-treated group, hepatocytes exhibited improved ultrastructural features compared with the AILI group, although some irregular nuclear morphology persisted. Mitochondria showed largely intact structures with more clearly defined cristae. Notably, multiple autolysosomes were observed in the cytoplasm, some of which appeared to contain degraded mitochondria. In the SIL + 3-MA group, hepatocytes displayed irregular morphology and nuclear changes. Mitochondria remained structurally intact but exhibited blurred cristae, and only a limited number of autolysosomes were present (Fig. 9A, B, C, D).

Quantitative TEM analysis further supported these observations. Compared with the control group, the AILI group showed a significant increase in the percentage of damaged mitochondria, accompanied by a significant decrease in autolysosome numbers. Following SIL treatment, the percentage of damaged mitochondria was significantly reduced, whereas autolysosome numbers were markedly increased. However, co-treatment with 3-MA partially attenuated the protective effects of SIL, as evidenced by an increased proportion of damaged mitochondria and a reduced number of autolysosomes relative to the SIL group (Fig. 9C, D).

## Discussion

Previous studies have demonstrated that silibinin attenuates toxin-induced liver injury by reducing oxidative stress and mitigating inflammatory responses [9, 27, 28]. However, the specific molecular mechanisms underlying these protective effects, particularly in the context of AILI, remain incompletely understood. A pivotal emerging concept in hepatoprotection is the role of mitophagy, a selective form of autophagy responsible for the removal of damaged mitochondria [29]. Given that mitochondrial dysfunction is a central driver of AILI—characterized by excessive ROS production, ATP depletion, and hepatocyte death—the accumulation of dysfunctional mitochondria represents a critical pathogenic event [17, 30–32].

In the present study, we systematically investigated the protective effects of silibinin against AILI using both in vitro and in vivo models, with a particular emphasis on autophagy regulation. Our findings provide novel mechanistic evidence that silibinin markedly attenuates hepatocyte injury by activating PINK1/Parkin–mediated mitophagy, thereby preserving mitochondrial integrity and promoting cellular recovery.

This study has several notable strengths. First, we demonstrated that silibinin markedly improved hepatocyte viability following AILI. This observation is in line with previous studies showing that silibinin exerts robust antioxidant and hepatoprotective effects across diverse models of liver injury, including toxin-induced damage and ischemia–reperfusion injury [15, 33–35]. Importantly, the observed restoration of hepatic SOD activity and reduction in MDA levels in our study suggests that silibinin effectively attenuates oxidative stress and mitochondrial dysfunction, which are key contributors to acetaminophen-induced hepatotoxicity [36, 37].

Second, our data revealed that silibinin effectively alleviated AILI-induced impairment of autophagy. This effect was evidenced by increased LC3-II levels accompanied by reduced p62 accumulation, suggesting restoration of autophagic flux rather than mere autophagosome accumulation. The enhancement of autophagic activity was associated with improved oxidative stress parameters, including elevated SOD levels and reduced MDA levels, supporting the notion that silibinin facilitates the clearance of damaged mitochondria. Given that impaired autophagy has been increasingly recognized as a critical contributor to AILI pathogenesis [38, 39], our findings highlight the therapeutic potential of targeting autophagy in this context.

Third, we identified the PINK1/Parkin signaling pathway as an important mediator of silibinin-induced mitophagy. Although AILI alone triggered upregulation of PINK1 and Parkin—likely as a compensatory response to mitochondrial damage—this adaptive response was insufficient to complete effective mitophagy, as indicated by p62 accumulation and persistent mitochondrial ultrastructural abnormalities. Notably, silibinin treatment further enhanced PINK1 and Parkin expression while simultaneously clearing p62 accumulation and restoring autophagic flux. These findings suggest that silibinin not only activates mitophagy-related signaling but also promotes the completion of the mitophagic process, thereby maintaining mitochondrial quality control. Importantly, genetic knockdown of PINK1 partially attenuated the protective effects of silibinin on autophagy and cell viability, providing evidence that the PINK1/Parkin axis is critical for silibinin-mediated hepatoprotection.

Most importantly, our results demonstrate that the hepatoprotective effect of silibinin extends beyond its well-known antioxidant and anti-inflammatory properties. We found that silibinin also promotes hepatocyte self-repair and regeneration through the activation of autophagy, thereby broadening the current understanding of its pharmacological actions. These findings suggest that silibinin may provide more comprehensive hepatic protection by regulating cellular energy metabolism and promoting hepatocyte proliferation.

Nevertheless, several limitations of the present study should be acknowledged. First, although we identified the PINK1/Parkin pathway as a central mechanism, we cannot exclude the involvement of other mitophagy-related pathways, such as BNIP3, FUNDC1, or SIRT1-mediated mitophagy [40–42]. Future studies employing pathway-specific inhibitors or genetic manipulation approaches could help delineate the relative contributions and potential crosstalk among these mitophagy pathways.

Second, the upstream regulatory mechanisms by which silibinin activates the PINK1/Parkin axis remain to be fully elucidated. In particular, the potential involvement of energy-sensing kinases, such as AMPK, or other metabolic regulators warrants further investigation. Elucidating these upstream signaling events would provide a more comprehensive understanding of how silibinin integrates metabolic stress signals to regulate mitophagy.

Third, although both in vitro and in vivo AILI models were employed, the translational relevance of our findings to human AILI patients remains to be validated. Future studies using human liver organoids or clinical samples could further strengthen the clinical applicability of our findings and facilitate the development of silibinin-based therapeutic strategies for drug-induced liver injury.

Building on our findings, several important directions emerge for future investigation. First, the interplay between autophagy and other stress-response pathways, such as endoplasmic reticulum stress, apoptosis, and inflammatory signaling, warrants deeper exploration to reveal how silibinin orchestrates hepatocyte survival and regeneration under AILI. Second, systematic studies on the pharmacokinetics and optimal dosing of silibinin are essential, particularly in models that closely mimic human AILI, to facilitate clinical translation. Third, integrating multi-omics approaches—including transcriptomics, proteomics, and metabolomics—could uncover comprehensive molecular networks regulated by silibinin and identify potential biomarkers for therapeutic efficacy and patient stratification. Finally, well-designed clinical studies, including early-phase trials in individuals at risk of acetaminophen-induced liver injury or with pre-existing hepatic impairment, are needed to validate the translational relevance of these preclinical findings. Collectively, pursuing these avenues will not only refine our mechanistic understanding of hepatoprotective actions of silibinin but also accelerate its rational development as a clinically applicable liver-protective therapy.

## Conclusion

In summary, our study demonstrates that silibinin exerts an impressive hepatoprotective effect against AILI. Silibinin not only restores hepatocyte viability but also effectively improves AILI-induced suppression of autophagy. Mechanistically, silibinin activates mitophagy through the PINK1/Parkin signaling pathway, thereby promoting mitochondrial quality control, enhancing hepatocyte survival and proliferation, and improving liver function. Collectively, these findings confirm silibinin as a promising therapeutic candidate for AILI and emphasize the critical role of mitophagy as a potential target in hepatoprotection.

## Data Availability

Data are available from the corresponding author on reasonable request.
